# Rapid isolation of gene homologs across taxa: Efficient identification and isolation of gene orthologs from non-model organism genomes, a technical report

**DOI:** 10.1186/2041-9139-2-7

**Published:** 2011-03-01

**Authors:** Alison Heffer, Leslie Pick

**Affiliations:** 1 Program in Molecular & Cell Biology and Department of Entomology, University of Maryland, 4112 Plant Sciences Building, College Park, MD 20742, USA

## Abstract

**Background:**

Tremendous progress has been made in the field of evo-devo through comparisons of related genes from diverse taxa. While the vast number of species in nature precludes a complete analysis of the molecular evolution of even one single gene family, this would not be necessary to understand fundamental mechanisms underlying gene evolution if experiments could be designed to systematically sample representative points along the path of established phylogenies to trace changes in regulatory and coding gene sequence. This isolation of homologous genes from phylogenetically diverse, representative species can be challenging, especially if the gene is under weak selective pressure and evolving rapidly.

**Results:**

Here we present an approach - Rapid Isolation of Gene Homologs across Taxa (RIGHT) - to efficiently isolate specific members of gene families. RIGHT is based upon modification and a combination of degenerate polymerase chain reaction (PCR) and gene-specific amplified fragment length polymorphism (AFLP). It allows targeted isolation of specific gene family members from any organism, only requiring genomic DNA. We describe this approach and how we used it to isolate members of several different gene families from diverse arthropods spanning millions of years of evolution.

**Conclusions:**

RIGHT facilitates systematic isolation of one gene from large gene families. It allows for efficient gene isolation without whole genome sequencing, RNA extraction, or culturing of non-model organisms. RIGHT will be a generally useful method for isolation of orthologs from both distant and closely related species, increasing sample size and facilitating the tracking of molecular evolution of gene families and regulatory networks across the tree of life.

## Findings

One focus of evolutionary biologists is to understand how changes in regulatory and coding regions of genes contribute to species evolution and adaptation [1,2]. This requires sequence comparisons across distantly related taxa as well as among closely related species. A major limitation in studying molecular evolution is the amount of comprehensive sequence data available to track these changes in genes and their networks. Standard approaches include comparisons across widely divergent model organisms, comparison of gene sequences that have been deposited in databases, and comparisons of whole genome sequences. This can result in an incomplete matrix of information about the lineages of particular gene families, making it difficult to trace steps leading to functional changes in regulatory and coding sequences. Additionally, the sequence conservation of duplicated and diverged genes within gene families [3,4] poses a challenge: How can we identify a particular member of a gene family without isolating and screening through closely-related homologs? Here we report a strategy to efficiently isolate genes from genomic DNA that can be used to obtain sequence information from un-sequenced genomes and non-model organisms not easily reared in the laboratory. Rapid Isolation of Gene Homologs across Taxa (RIGHT) is based on the fact that homologous genes (both paralogs and orthologs) generally show conservation of at least one domain, even if other parts of the sequence are under weaker selective pressure. For example, the Hox proteins have retained the conserved DNA binding domain after duplication and divergence [5,6]. While not forging fundamentally new technology, this approach combines and modifies existing procedures to facilitate the rapid isolation of genes, allowing sampling of a large number of taxa.

### RIGHT Methodology

**Figure 1:**
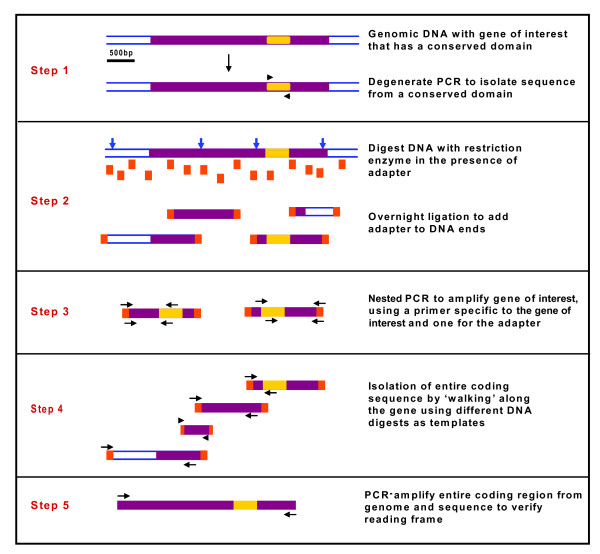
**Overview of RIGHT technique used to isolate homologous genes from large gene families**. All steps are described in the text. Oligonucleotides that were annealed to make the adapter destroyed the restriction site. All reverse primers were ordered with the 5' end phosphorylated, or were phosphorylated before annealing with an appropriate enzyme. For example, *MseI *digest/ligation: F-5' GACGATGAGTCTTGAGTTCAGTCTGTA, R-5'PhosTATACAGACTGAACTCAAGACTCATC; *XhoI*: F-5' GACGATGAGTCTTGAGTTCAGTCTGTA, R-5'PhosTCGATACAGACTGAACTCAAGACTCATC

RIGHT methodology utilizes degenerate polymerase chain reaction (PCR) and gene-specific amplified-length fragment polymorphism (AFLP) to allow for rapid gene isolation. First, degenerate primers are designed to amplify a small region of less than 200 bases of the conserved domain characteristic of the gene family (Figure 1, Step 1). One primer is derived from the unique signature motif in the homolog of interest, while the other can be shared with other family members. A variation of touchdown PCR is used that is optimized for these degenerate primers (see attached protocol, Additional file 1). Nested PCR is done to ensure that the correct gene-family member is amplified. The PCR product is run on an agarose gel, purified and sequenced. The product can be positively identified by characteristic residues in the homolog of interest, along with BLAST searches against other species. Together, this allows for increased confidence that the isolated gene product corresponds to the gene of interest. From this short DNA sequence obtained from degenerate PCR, gene-specific primers are designed for subsequent reactions.

Sequence up- and downstream of the conserved region (obtained in Step 1, Figure 1) is next isolated by modifications of AFLP and TE-display techniques [7-12] that allow selective amplification of only the gene sequence of interest. Traditional AFLP uses restriction enzymes to digest genomic DNA followed by ligation of adapters of known sequence to DNA ends. Adapter-specific primers are used in subsequent PCRs to amplify DNA fragments, which are then separated on a gel and analyzed. RIGHT uses the basic idea of AFLP up to the amplification step; however, instead of amplifying DNA fragments using adapter sequences as both primers (which generates many fragments), an adapter-specific primer is used as one primer and a gene-specific primer (derived from degenerate PCR used in Figure 1, Step 1) as the other primer. Thus, only a sequence from the gene of interest is isolated. The digestion of genomic DNA and ligation of adapters is done in a single step (Figure 1, Step 2). Adapter sequences are designed to anneal to, but destroy, restriction sites in order to avoid re-digestion in this combined restriction/ligation reaction. Several different restriction digests are set up in parallel to provide different-length PCR templates covering the gene of interest. This is also beneficial because restriction site locations are not known for genomes that have not been sequenced. The digestion/ligation is followed by two rounds of nested PCR (Figure 1, Step 3), which functions to increase specificity of primer binding and the amount of product. After the PCR product is amplified and sequenced, new gene-specific primers are designed at the sequence ends to repeat PCRs with a different restriction digest/ligation as template in order to extend the sequence. By repeating this process, one can "walk" along the genomic sequence to isolate the entire coding sequence (Figure 1, Step 4).

In most cases only one clear product was observed after nested PCR; however, occasionally there were several. In these situations, either all products were sequenced or products were re-amplified using the same primers or another nested set to reduce the number of products. In cases where multiple bands persisted, it was usually due to restriction sites that were very close together in the genome and almost all of the sequenced regions overlapped. After a new sequence has been isolated, its continuity is always checked by PCR with primers at extreme opposite ends of the sequence that has been obtained to make sure the sequence being isolated is contiguous with that upstream and/or downstream (Figure 1, Step 5). This is very important because, although infrequent, ligation may occur between genomic DNA fragments in Step 2. As demonstrated, RIGHT provides efficiency and saves time when compared to other protocols. This combination is a powerful method for obtaining full gene sequence information, including coding and regulatory regions.

### RIGHT isolation of homeobox and nuclear receptor genes

**Figure 2:**
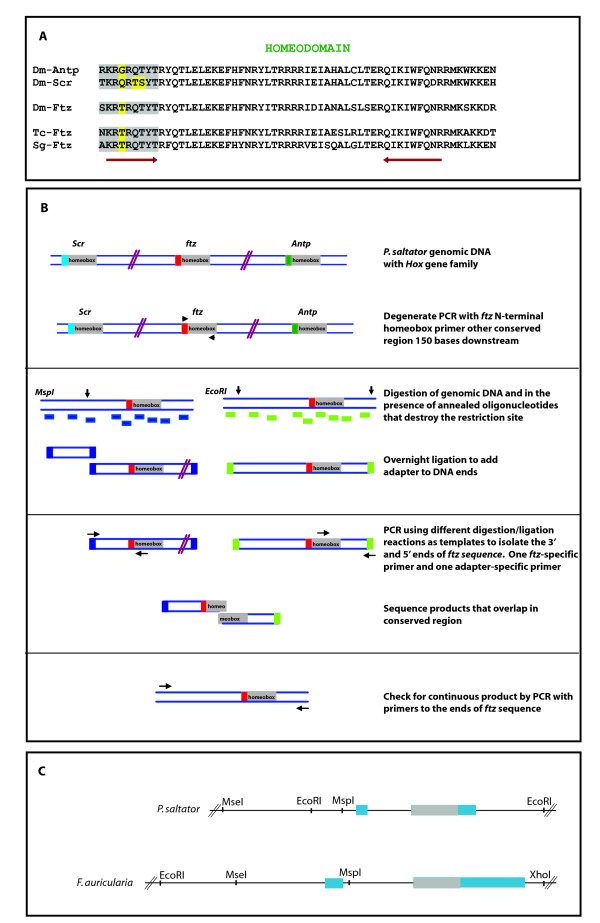
**Isolation of *ftz *homologs using RIGHT**. **A) **Homolog-specific residues guide degenerate PCR design. Different Hox proteins have different N-terminal regions (grey shaded region, with differences highlighted in yellow) that can be used for isolation of one family member. The arrows indicate the regions used for degenerate primer design to isolate *ftz*. The forward degenerate primer makes use of the signature motifs in the N-terminal region, allowing for specific amplification of one member of the *Hox *gene family. (*Drosophila melanogaster *Antp: Dm-Antp; *D. melanogaster *Scr: Dm-Scr; *D. melanogaster *Ftz: Dm-Ftz; *Tribolium castaneum *Ftz: Tc-Ftz; *Schistocerca gregaria *Ftz: Sg-Ftz). **B) **Isolation of *ftz *from genomic DNA of non-model organism. A schematic of one application of our approach to isolate new homologs is shown. **C) **Degenerate PCR was used to isolate the *ftz *homebox of P.salt and F.auri, and full-length *ftz *sequences were obtained using different restriction digests/ligations and subsequent PCRs. For *P. salt*, three fragments were obtained by RIGHT and sequenced after degenerate PCR identified the *ftz *homeobox; fragment sizes are (from left to right): *MseI-EcoRI *320 bp, *EcoRI-MspI *114 bp, *MspI-EcoRI *945 bp. For *F. auri*, three fragments were also obtained by RIGHT and sequenced after degenerate PCR identified the *ftz *homeobox; fragment sizes are (from left to right): *EcoRI-MseI *273 bp, *MseI-MspI *383 bp, *MspI-XhoI *875 bp. Homeobox regions are shown in grey, and other coding regions in blue.

RIGHT has been used successfully in our laboratory to efficiently isolate specific members of several large gene families. The technique was first developed to isolate a rapidly-evolving member of the *Hox *gene family, *fushi tarazu *(*ftz) *[13-15]. First, degenerate PCR primers were designed based upon signature residues encoding the amino-terminal end of the DNA-binding homeodomain and another highly conserved region with low degeneracy approximately 150 bases downstream (Figure 2A, arrows). Ftz homologs were positively identified by characteristic residues in the homeodomain. Next, gene-specific AFLP was carried out using a combination of unique restriction/ligation templates for PCR with one *ftz*-specific primer and one adapter-specific primer (Figure 2B). All products were sequenced and gene-sequence continuity verified by PCR with genomic DNA and primers designed to the extreme 5' and 3' ends of sequence that had been isolated. Full-length *ftz *sequences, including putative introns, were isolated by genomic walking until translation initiation and stop codons were identified. Using RIGHT allowed us to isolate *ftz *genes from diverse arthropods representing approximately 550 million years of evolutionary divergence [16], including the dermapteran *Forficula auricularia *and archaeognathan *Pedetontus saltator *(Figure 2C). Additionally, genomic DNA of two non-model beetles was used for degenerate PCR to obtain the *ftz *homeobox, and in combination with RACE on embryonic cDNA, full-length *ftz *sequences were obtained [16]. To date, we have isolated 2 full-length and 10 partial *ftz *genes from a range of non-model organisms using RIGHT.

In addition to *ftz*, we isolated other homeobox-containing genes such as *extradenticle *(*exd*) and the orphan nuclear receptor *ftz-f1 *from multiple species with great success (unpublished). RIGHT was used to isolate partial *exd *sequences from *Thermobia domestica *(firebrat), *Callosobruchus maculatus *(beetle), and *Folsomia candida *(collembolan). In combination with RACE, full-length *exd *coding regions were isolated from these species. Several partial *ftz*-*f1 *sequences were isolated, including *Artemia salina *(brine shrimp)*, Folsomia candida *(collembolan), *Thermobia domestica *(firebrat), *Callosobruchus maculatus *(beetle), *Dermestes maculatus *(beetle) *Oncopeltus fasciatus *(milkweed bug), and *Acyrthosiphon pisum *(aphid). As for *exd*, full-length *ftz-f1 *sequences have been obtained from many of these organisms in combination with RACE. For this work, as per experimental design, sequences were obtained from species representing key points in arthropod phylogeny to allow for systematic analysis of a small network of functionally related genes from different families (*ftz, ftz-f1, exd*). Thus far, every gene that we have attempted to isolate from any chosen species using RIGHT has been obtained.

## Conclusions

The ability to isolate homologous genes from diverse taxa will empower studies of molecular evolution of genes, families and gene networks. In the past, these approaches were limited by absence of genomic information. Even though genome sequencing is now practical for a larger number of species, it is unlikely to make a dent in the millions of species on Earth. Similarly, investments are being made in developing new model systems, to expand on the standard fly, mouse and worm systems. However, the investment to bring a new model system up to speed is substantial and it is neither necessary nor practical to fully develop hundreds of genetic model systems. We suggest that these approaches, while enormously important for the field of evo-devo, are not always necessary to answer specific evolutionary questions. RIGHT provides a fast and efficient way to isolate genes, including coding regions and candidate cis-regulatory regions, and overcomes many practical constraints, realistically allowing for the isolation of 10s if not 100s of genes from families or gene networks to study molecular evolution across divergent taxa or within specific clades. This approach obviates common limitations, such as genome sequence availability or rearing species in the lab. It has been used successfully to isolate specific members of several large gene families, allowing for a comparative analysis over millions of years of evolutionary time.

## Abbreviations

AFLP: Amplified Fragment Length Polymorphism; *Antp*: *Antennapedia*; *exd*: *extradenticle*; *ftz*: *fushi tarazu*; *ftz-f1*: *fushi tarazu factor 1*; PCR: Polymerase Chain Reaction; RACE: Rapid Amplification of cDNA Ends; RIGHT: Rapid Isolation of Gene Homologs across Taxa; *Scr*: *Sex combs reduced*; TE-display: Transposable Element-display

## Competing interests

The authors declare that they have no competing interests.

## Authors' contributions

AH participated in experimental design, carried out experiments and participated in writing the paper. LP participated in experimental design, analysis of results and writing the paper.

## Supplementary Material

Additional file 1 **Heffer and Pick Supplementary Information Protocol**. A detailed description of protocol for the technique described in this manuscript. Click here for file

## References

[B1] Schlosser G Wagner GP Modularity in Development and Evolution 2004 Chicago: University of Chicago Press

[B2] Carroll SB Grenier JK Weatherbee SD From DNA to diversity: molecular genetics and the evolution of animal design 2005 Second Oxford: Blackwell Science Ltd

[B3] Ohno S Evolution by gene duplication 1970 Berlin: Springer-Verlag

[B4] Lynch M Force A The probability of duplicate gene preservation by subfunctionalization Genetics 2000 154 459 473 10629003 10.1093/genetics/154.1.459 PMC1460895

[B5] McGinnis W Krumlauf R Homeobox genes and axial patterning Cell 1992 68 283 302 10.1016/0092-8674(92)90471-N 1346368

[B6] Gehring WJ Kloter U Suga H Evolution of the Hox gene complex from an evolutionary ground state Curr Top Dev Biol 2009 88 35 61 full_text 19651301 10.1016/S0070-2153(09)88002-2

[B7] Vos P Hogers R Bleeker M Reijans M van de Lee T Hornes M Frijters A Pot J Peleman J Kuiper M AFLP: a new technique for DNA fingerprinting Nucleic Acids Res 1995 23 4407 4414 10.1093/nar/23.21.4407 7501463 PMC307397

[B8] Beeman RW Stauth DM Rapid cloning of insect transposon insertion junctions using 'universal' PCR Insect Mol Biol 1997 6 83 88 10.1046/j.1365-2583.1997.00159.x 9013259

[B9] Casa AM Brouwer C Nagel A Wang L Zhang Q Kresovich S Wessler SR Inaugural article: the MITE family heartbreaker (Hbr): molecular markers in maize Proc Natl Acad Sci USA 2000 97 10083 10089 10.1073/pnas.97.18.10083 10963671 PMC27704

[B10] Biedler J Qi Y Holligan D della Torre A Wessler S Tu Z Transposable element (TE) display and rapid detection of TE insertion polymorphism in the Anopheles gambiae species complex Insect Mol Biol 2003 12 211 216 10.1046/j.1365-2583.2003.00403.x 12752653

[B11] Hawthorne DJ AFLP-based genetic linkage map of the Colorado potato beetle Leptinotarsa decemlineata: sex chromosomes and a pyrethroid-resistance candidate gene Genetics 2001 158 695 700 11404333 10.1093/genetics/158.2.695 PMC1461671

[B12] Subramanian RA Arensburger P Atkinson PW O'Brochta DA Transposable element dynamics of the hAT element Herves in the human malaria vector Anopheles gambiae s.s Genetics 2007 176 247724 87 10.1534/genetics.107.071811 PMC1950647 17603116

[B13] Telford MJ Evidence for the derivation of the Drosophila fushi tarazu gene from a Hox gene orthologous to lophotrochozoan Lox5 Curr Biol 2000 10 349 352 10.1016/S0960-9822(00)00387-0 10744975

[B14] Lohr U Yussa M Pick L Drosophila fushi tarazu: a gene on the border of homeotic function Curr Biol 2001 11 1403 1412 10.1016/S0960-9822(01)00443-2 11566098

[B15] Lohr U Pick L Cofactor-interaction motifs and the cooption of a homeotic Hox protein into the segmentation pathway of Drosophila melanogaster Curr Biol 2005 15 643 649 10.1016/j.cub.2005.02.048 15823536

[B16] Heffer A Shultz J Pick L Surprising flexibility in a conserved Hox transcription factor over 550 million years of evolution Proc. Natl. Acad. Sci. USA 2010 107 18040 18045 10.1073/pnas.1010746107 PMC2964257 20921393

