# Supplementary material for: Rapid isolation of gene homologs across taxa: Efficient identification and isolation of gene orthologs from non-model organism genomes, a technical report
Source: EvoDevo. 2011 Mar 1;2:7. doi: 10.1186/2041-9139-2-7 (PMC3055837; doi:10.1186/2041-9139-2-7)
Supplement: Additional file 1 — Heffer and Pick Supplementary Information Protocol. A detailed description of protocol for the technique described in this manuscript. [file 2041-9139-2-7-S1.PDF]

RIGHT Protocol

1. Degenerate PCR (1-2 rounds of PCR)
   a) Use 100-500ng genomic DNA as template
   b) Since primers are degenerate, use a "touchdown PCR" program to amplify fragments

      94^oC 5 min
      Cycle 24 times:
      94^oC 30 sec
      *50^oC 30 sec, -0.4^o each cycle
      72^oC 30 sec, + 2 sec each cycle
      Cycle 12 times:
      94^oC 30 sec
      40^oC 40 sec
      72^oC 1 min, + 3 sec each cycle
      Final extension:
      72^oC 5 min
      4^oC forever

   b) Run entire reaction on 2% agarose gel to check for product
   c) Extract product(s) from gel, purify and sequence.
   d) Design gene-specific primers to amplify further sequence

2. Digestion of genomic DNA
   a) Measure concentration of DNA on spectrophotometer; use 500 ng-1.5 µg total genomic DNA in each digest
   b) Set up 40 µl digests: DNA, 5 µl appropriate buffer, 0.5 µl BSA; 10U enzyme (4 base-pair cutter or 6 base-cutter)
   c) Incubate at 37^oC while oligonucleotides are annealing (See 2 below; ~35-40 minutes)

3. Anneal oligonucleotides to make the adapter
   a) Recommended adapter concentration is 50 pmol** (ex: if stock primer is 100 µM, use 0.5 µl each oligo)
   b) In PCR tube, set up annealing reaction: 50 pmol each oligonucleotide, 1X annealing buffer (100 mM Tris pH 7.9, 10 mM $MgCl_2$), to 4.75 µl with H_2O
   c) Anneal oligonucleotides in thermocycler:

      88^oC 2 min
      65^oC 10 min
      37^oC 10 min
      25^oC 10 min

*Depending on degenerate primer annealing temperatures, this starting temperature may have to be changed
**Ligation reaction is more efficient if the reverse adapter oligonucleotide is phosphorylated

4. Ligation reaction
   a) Add to annealed oligonucleotides: 1µl T4 DNA Ligase Buffer (New England Biolabs)
      DTT (final concentration of 5mM)
      ATP (final concentration of 2mM)
   b) Add all of ligation mixture to digest, then add 1.75 µl T4 DNA ligase to entire reaction
   c) Incubate digestion/ligation overnight at room temperature

5. PCR amplification (2 rounds of PCR)
   a) Use 5 µl of digest/ligation as a template (~50 ng) in a 20 µl reaction; primer concentrations of 10 pmol each.
   b) Use appropriate PCR program for gene-specific primers
   c) Dilute reactions by adding 100 µl 10 mM Tris pH 7.9, 10 mM $MgCl_2$
   d) Use 2 µl diluted PCR product as a template in a 20 µl reaction. All other concentrations remain the same.
   e) Use appropriate PCR program for gene-specific primers to amplify fragment
   f) Run entire reaction on 2% agarose gel to check for products.
   g) Extract product(s) from gel, purify and sequence. More than one product may result from amplification of more than one product due to restriction sites being close in proximity, or poor primer design.
